# Characterising Through-Thickness Shear Anisotropy Using the Double-Bridge Shear Test and Finite Element Model Updating

**DOI:** 10.3390/ma18102220

**Published:** 2025-05-11

**Authors:** Bojan Starman, Bin Chen, Andraž Maček, Yi Zhang, Miroslav Halilovič, Sam Coppieters

**Affiliations:** 1 Faculty of Mechanical Engineering, University of Ljubljana, Aškerčeva 6, 1000 Ljubljana, Slovenia; bojan.starman@fs.uni-lj.si (B.S.); andraz.macek@fs.uni-lj.si (A.M.); 2 Department of Materials Engineering, KU Leuven, Ghent Campus, 9000 Ghent, Belgium; yizhang@ustb.edu.cn; 3 Wallenberg Wood Science Center, Department of Fiber and Polymer Technology, KTH Royal Institute of Technology, 10044 Stockholm, Sweden; binchen@kth.se; 4 Collaborative Innovation Center of Steel Technology, University of Science and Technology Beijing, Beijing 100083, China

**Keywords:** plastic anisotropy, shear testing, material identification, digital image correlation, Finite Element Model Updating

## Abstract

The accuracy of numerical predictions in sheet metal processes involving multiaxial stress–strain states (e.g., blanking, riveting, and incremental forming) heavily depends on the characterisation of plastic anisotropy under multiaxial loading conditions. A fully calibrated 3D plastic anisotropy model is essential for this purpose. While in-plane material behaviour can be conventionally characterised through uniaxial and equi-biaxial tensile tests, calibrating out-of-plane material behaviour remains a significant challenge. This behaviour, governed by out-of-plane shear stress and associated material parameters, is typically described by out-of-plane shear yielding. These parameters are notoriously difficult to determine, leading researchers to frequently assume isotropic behaviour or identical shear parameters for in-plane and out-of-plane responses. Although advanced calibrations may utilise crystal plasticity modelling, there remains a critical need for macro-mechanical characterisation methods. This paper presents an out-of-plane shear testing and material characterisation procedure based on full-field strain measurements using digital image correlation (DIC). Strains within the shear zone are measured via DIC and employed in the Finite Element Model Updating (FEMU) to identify out-of-plane shear parameters of a 2.42 mm thick, cold-rolled AW5754-H22 aluminium alloy sheet, using the Yld2004-18p yield criterion. Given that the characteristic strain response at this scale may be influenced by local crystal structure behaviour on the surface, this paper evaluates the feasibility of such measurements. Finally, to test the validity of the full-field-based approach, the FEMU-identified parameters are compared against results obtained through a classical optimisation procedure based on force-elongation measurements from the shear zone.

## 1. Introduction

The development of modern engineering products increasingly relies on advanced simulation tools, which are essential for creating accurate numerical models of technological processes. These models enable engineers to analyse manufacturing methods and optimise technological parameters prior to production. For sheet metal processing, numerical models—typically based on the finite element method (FEM)—are significantly influenced by the material model used. Accurately representing physical behaviour necessitates incorporating phenomena like elasto-plasticity with hardening and plastic anisotropy. While traditional forming operations typically employ plane stress models with simplified anisotropy descriptions, as outlined by Banabic et al. [1], more complex processes like riveting [2,3], flow forming [4], incremental forming [5,6], rolling [7], blanking [8], and ironing involve multiaxial stress states. For these, multiaxial yield functions are crucial.

Plane stress yield functions can be calibrated using standard biaxial and uniaxial tensile tests using rolling (RD), transverse (TD), and diagonal (DD) directions. Yield functions addressing general stress states require additional calibration for out-of-plane shear responses. The Hill48 yield function [9], for instance, uses parameters *F*, *G*, *H*, and *N* for in-plane stresses, and *L* and *M* for thickness shear. As suggested by Barlat et al. [10], when direct measurements of out-of-plane shear are impractical, crystal plasticity modelling or assuming an isotropic out-of-plane shear equivalent to an in-plane response can be alternatives. The importance of modelling out-of-plane shear is highlighted in studies by Esmaeilpour et al. [11], who compared yield functions for incremental forming simulations, assigning isotropic shear due to a lack of data. Subsequent works [12,13] validated crystal plasticity-generated synthetic data experimentally.

When crystal plasticity modelling is not feasible, assuming isotropic shear response remains an option, though it is less robust than macroscopic mechanical characterisation. Shear testing, traditionally significant for characterising large strain hardening [14] and reverse loading [15], faces challenges in generating sensitive through-thickness shear data. However, generating an out-of-plane shear stress state through macro-mechanical testing presents notable challenges, particularly in achieving high sensitivity of measured response to through-thickness shear yield stress variations.

In contrast to out-of-plane testing, numerous experimental methods and test specimens have been devised to investigate in-plane shear behaviour. Miyauchi’s pioneering work [16] introduced a method that applies longitudinal displacement to opposing sides of a rectangular specimen, producing a simple shear state. This methodology has been widely adopted, with researchers such as Bouvier et al. [17] examining strain-induced plastic anisotropy and Carbonniere et al. [18] comparing work hardening under bending–unbending and simple shear conditions. Similarly, An et al. [19] demonstrated that rectangular specimens provide relatively uniform stress fields for work hardening investigations. One of the key advantages of Miyauchi’s approach is its ability to derive stress–strain relationships analytically from force–extension data, provided that the shear zone maintains a homogeneous stress–strain field [20]. To ensure this condition, Bouvier et al. [17] recommended maintaining a length-to-width ratio of greater than 10. Despite its utility, Miyauchi’s method is mechanically complex due to the moments and high transverse forces generated during testing [21]. To address these issues, Zillmann et al. [22] proposed a specimen design featuring slits that divide the specimen into three strips linked by shear zones, thereby mitigating adverse moments and simplifying the testing process. Likewise, Yin et al. [23] developed a cyclic twin-bridge shear specimen, utilising an in-plane twist test that ensures uniform shear zone behaviour and is particularly suited for cyclic loading studies. Alternative specimen designs following ASTM standards [24] offer additional solutions, often requiring minimal specialised equipment. For example, the V-notched shear specimen, initially proposed by Iosipescu [25], has become a standard tool despite its tendency to rotate under load due to inadequate stiffness in the surrounding material. Researchers [26,27] have countered this limitation by introducing design modifications, such as reinforcing the specimen or incorporating custom shear details to investigate plastic flow under complex strain paths.

While considerable advancements have been made in in-plane shear testing, methods for the through-thickness shear characterisation of sheet metals remain underdeveloped. Reviews by Ma and Wang [28] highlight the reliance on specialised equipment and the susceptibility of existing methods to frictional interference. A noteworthy exception is the notch-based shear specimen developed by Li et al. [29], Han et al. [30], which employs a universal testing machine without requiring bespoke apparatus. Nevertheless, this approach is limited by shear zone rotation under large strains and inherent differences between in-plane and through-thickness stress states.

Recent efforts have focused on overcoming these limitations through innovative experimental setups and analytical methods. For instance, Lattanzi et al. [31] adapted the Iosipescu shear test for sheet specimens, combining digital image correlation (DIC) with Finite Element Model Updating (FEMU) [32] to calibrate parameters for the Hill48 anisotropy model. Similarly, in [33], the authors employed a hybrid approach, integrating a Virtual Field Method (VFM) for in-plane anisotropy characterisation with FEMU for through-thickness analysis. The authors of Hakoyama et al. [34] introduced a tensile test configuration designed to induce through-thickness shear in strip specimens, though challenges such as large rotations and low shear strains persisted. A comprehensive review by Han et al. [35] underscores the challenges associated with calibrating out-of-plane shear parameters in advanced yield criteria. They note that the immaturity of current shear test methods hinders progress, and they advocate for the adoption of emerging machine learning techniques to enhance parameter calibration. The main reason for this is the low sensitivity of the measurement to the constitutive parameters in the current test design. This growing field of research underscores the pressing need for robust methodologies to characterise through-thickness shear behaviour in sheet metals.

A novel macro-mechanical methodology for characterising the out-of-plane shear behaviour of anisotropic sheet materials was proposed by Satošek et al. [36]. This approach employed a two-stage calibration procedure to fully determine the Yld2004-18p yield criterion. To calibrate the out-of-plane shear parameters, the authors used an instrumented ball indentation test, which resulted from the anisotropic behaviour of the sheet in a non-axisymmetric pile-up profile. This profile was then utilised in a FEMU-U (FEMU using displacement field information in the cost function) inverse identification scheme to determine the out-of-plane shear parameters. Notably, as this procedure relies on the geometry of the residual imprint, it does not capture the actual evolution of strain fields beneath the indenter, which can hardly be measured using optical DIC-based methods.

To address the challenges of out-of-plane shear response characterisation, we recently developed an innovative shear specimen geometry with identical shear detail dimensions for both in-plane and out-of-plane shear testing [37]. This specimen design facilitates direct comparisons of shear responses across different material planes. Moreover, the test exhibits significant sensitivity of the force–extension response to variations in the Yld2004-18p out-of-plane parameters, which are identified using a FEMU-F (FEMU using force information in the cost function) approach. Although the shear response was analysed using DIC, it was not employed for parameter identification. Instead, DIC served as a sanity check and played a critical role in identifying and mitigating systematic errors such as those caused by manufacturing inaccuracies, specimen handling, initial sheet curvature, clamping misalignment, and loading eccentricities, all of which can profoundly affect the measured strain fields. In this paper, we present a comprehensive experimental numerical procedure for identifying out-of-plane shear parameters using full-field measurements in combination with direct-levelling-based FEMU [38]. Compared to other studies [33,34], our specimen design enables the characterisation of differences in responses across various material planes due to the enhanced sensitivity of the measured force response. However, since the measured strain fields directly reflect the surface behaviour of the specimen, a pertinent research question arises whether the strain response characterises the shearing of the zone or represents the response of individual grains on the specimen’s surface. The paper is structured as follows. Section 2 briefly presents the theoretical background of the Yld2004-18p model, which is used to characterise the out-of-plane shear behaviour in combination with the direct-levelling-based FEMU. The out-of-plane shear testing procedure, including a description of the DIC setup and measurement, is detailed in Section 2.4. The entire methodology of the direct-levelling-based FEMU procedure is introduced in Section 2.5 and demonstrated through the concept of virtual experimentation in Section 3.1. Finally, the calibration of out-of-plane shear anisotropy (Section 3.3) follows the presentation of the measured shear test results in Section 3.2.

## 2. Materials and Methods

In this section, we revisit the formulation of the Yld2004-18p model, which is utilised for the characterisation of anisotropy. Due to the large number of material parameters requiring calibration in the yield function, this section focuses specifically on the parameters associated with out-of-plane shear behaviour. Parameters related to in-plane yielding behaviour are determined from conventional uniaxial and equi-biaxial tensile tests. The reduced number of unknown parameters can improve the calibration robustness for the parameters related to out-of-plane shear behaviour.

### 2.1. Yld2004-18p Model

In this study, the out-of-plane shear behaviour is modelled using the Yld2004-18p yield function, which is specifically designed to describe anisotropic plastic yielding under multiaxial stress state conditions. This yield model can better describe the sheet metal plasticity compared with the simpler models. However, more model parameters necessitate careful model calibration. A brief overview of the model formulation is provided in the following section. For a more detailed theoretical background, readers are encouraged to consult the original work by Barlat et al. [10].

The description of the plastic anisotropy is associated with a material coordinate system, with x, y, and z corresponding to three orthogonal material directions. The yield criterion of the model is defined as

(1) $$\Phi \left(\sigma ,\sigma_{y}\right)=\sum_{i=1}^{3}\sum_{j=1}^{3}{\left|{\tilde{S}}_{i}^{\prime }-{\tilde{S}}_{j}^{\prime \prime }\right|}^{a}-4\sigma_{y}^{a}=0.$$

In Equation (1), the values ${\tilde{S}}_{i}^{\prime }$ and ${\tilde{S}}_{j}^{\prime \prime }$, {i,j}∈{1,2,3}, are the eigenvalues of the deviatoric stress tensors ${\tilde{s}}^{\prime }$ and ${\tilde{s}}^{\prime \prime }$, respectively. The exponent *a* captures the impact of the crystallographic structure and is equal to 6 for Body-Centred Cubic (BCC) materials and 8 for Face-Centred Cubic (FCC) materials. The reference yield stress in Equation (1) is denoted by $\sigma_{y}$ and is a function of equivalent plastic strain $\varepsilon_{\mathrm{eq}}^{\mathrm{pl}}$ accumulated through the yielding process; it is defined by the isotropic hardening law. The plastic anisotropy is modelled through two fourth-order linear transformations applied to the Cauchy stress tensor $\sigma ={\left\{\sigma_{xx},\sigma_{yy},\sigma_{zz},\sigma_{xy},\sigma_{yz},\sigma_{zx}\right\}}^{T}$ as

(2) $${\tilde{s}}^{\prime }=C^{\prime }:s=C^{\prime }:T:\sigma =L^{\prime }:\sigma ,\;{\tilde{s}}^{\prime \prime }=C^{\prime \prime }:s=C^{\prime \prime }:T:\sigma =L^{\prime \prime }:\sigma ,$$

where $C^{\prime }$ and $C^{\prime \prime }$ present the fourth-order linear transformation tensors, defined as

(3) $$C^{\prime }=\left[\begin{array}{cccccc} 0 & -\alpha_{1} & -\alpha_{2} & 0 & 0 & 0\\ -\alpha_{3} & 0 & -\alpha_{4} & 0 & 0 & 0\\ -\alpha_{5} & -\alpha_{6} & 0 & 0 & 0 & 0\\ 0 & 0 & 0 & \alpha_{9} & 0 & 0\\ 0 & 0 & 0 & 0 & \alpha_{7} & 0\\ 0 & 0 & 0 & 0 & 0 & \alpha_{8} \end{array}\right],\;C^{\prime \prime }=\left[\begin{array}{cccccc} 0 & -\alpha_{10} & -\alpha_{11} & 0 & 0 & 0\\ -\alpha_{12} & 0 & -\alpha_{13} & 0 & 0 & 0\\ -\alpha_{14} & -\alpha_{15} & 0 & 0 & 0 & 0\\ 0 & 0 & 0 & \alpha_{18} & 0 & 0\\ 0 & 0 & 0 & 0 & \alpha_{16} & 0\\ 0 & 0 & 0 & 0 & 0 & \alpha_{17} \end{array}\right].$$

In the formulation above, the anisotropic behaviour is captured by 18 material parameters $\alpha_{i},\;i\in \left\{1,2,\ldots ,18\right\}$. If the parameters are equal to one, the model reduces to the isotropic case. Due to non-uniqueness, the number of parameters can be reduced to 16 [39], and without any loss of generality, $\alpha_{1}=\alpha_{2}=1$ can be assumed. Moreover, when $C^{\prime }=C^{\prime \prime }$, the model reduces to the Yld91 model [40].

Typically, twelve parameters associated with in-plane material behaviour (in the x−y plane) are identified from the uniaxial tension data from five directions between the rolling (RD or x) and transverse (TD or y) directions and the balanced biaxial test data. For the determination of the remaining 4 parameters associated with the out-of-plane shear behaviour in x−z and y−z material planes (i.e., ($\alpha_{7},\alpha_{8},\alpha_{16},\alpha_{17}$)), three options are viable when direct mechanical measurement of shear resistance is not feasible [10]: isotropic case (i.e., $\alpha_{7}=\alpha_{8}=\alpha_{16}=\alpha_{17}=1$), equal in- and out-of-plane parameters (i.e., $\alpha_{7}=\alpha_{8}=\alpha_{9}$ and $\alpha_{16}=\alpha_{17}=\alpha_{18}$), or using the CPFEM approach, as described in Section 1.

### 2.2. Chemical Composition and Microstructure of AW5754-H22

The material tested in this study is a 2.42 mm thick AW5754-H22 aluminium alloy sheet metal. During production, the material undergoes strain hardening through the rolling process, reaching 25% of its total strain-hardening capacity. This is followed by partial annealing, which relieves internal stresses while preserving some of the hardening effects. The chemical composition of the material can be found in [41].

Since the primary objective of this study is to characterise out-of-plane shear anisotropy as a direct consequence of variations in the crystal structure across different material planes, a crystallographic analysis was conducted at different material planes and different thicknesses to analyse the difference in crystal structure. The analysis was performed using a Scanning Electron Microscope (SEM, ZEISS Crossbeam 550 FIB-SEM Gemini II 6500F, Oberkochen, Germany) equipped with Energy Dispersive X-ray Spectroscopy (EDS, EDAX Octane Elite, Mahwah, NJ, USA) and Electron Backscatter Diffraction (EBSD, EDAX Hikari Super, Mahwah, NJ, USA). The microstructural analysis primarily focused on evaluating potential differences in grain size across various material planes: the x−y plane for the in-plane shear response and the y−z and x−z planes for the out-of-plane response. Additionally, due to the rolling process, through-thickness structural variations are expected to some extent. Therefore, the crystallographic analysis that measures the crystal structure at the nanoscale was conducted at two depths: at the centre of the sheet (1.21 mm below the surface) and at the location of the shear details, i.e., 0.8 mm below the surface.

**Figure 1:**
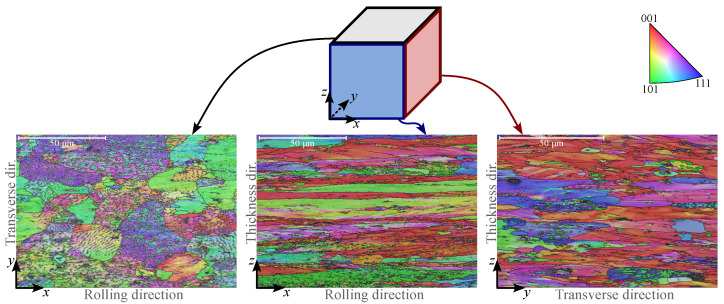
The crystallographic analysis of 2.42 mm thick AW5754-H22 sheet metal across three material planes. While the grain structure appears more equiaxed in the x−y plane, it is highly elongated in the rolling direction in the x−z plane. An elongation is also present in the y−z plane, but it is less pronounced [37]. (Reproduced with permission from https://www.sciencedirect.com/journal/international-journal-of-solids-and-structures, accessed on 6 May 2025).

The results, as presented in Figure 1, indicate that the grains in the x−y plane appear more equiaxed, whereas the grain structure along the rolling direction in the x−z plane is highly elongated. A similar elongation is also observed in the y−z plane (transverse direction), although it is less pronounced.

To evaluate how representative the shear resistance measurement is of the uniform behaviour across the sheet’s entire thickness, variations across the thickness were also analysed. The results show that the difference between the crystal structure in the middle of the sheet and 0.8 mm below the sheet’s surface is practically indistinguishable in the x−z plane. However, in the y−z plane, the grains appear slightly smaller at 0.8 mm below the surface.

### 2.3. Out-of-Plane Shear Test Specimens

The methodology presented in this study follows the concept of double-bridge shear in-plane testing, originally proposed by Zillmann et al. [22] for hardening characterisation at large strains beyond those measurable with standard uniaxial tests. While the single-bridge shear test [35] or the notch shear test [34] can also be used to characterise out-of-plane shear resistance, they have relatively poor sensitivity of the measured force to the material parameters and may be affected by shear zone rotation, which arises due to a force couple. The proposed shear test design has the key advantages of (1) enhanced sensitivity of the measured force response to variations in material parameters and (2) improved resistance to shear zone rotation and ligament deformation.

**Figure 2:**
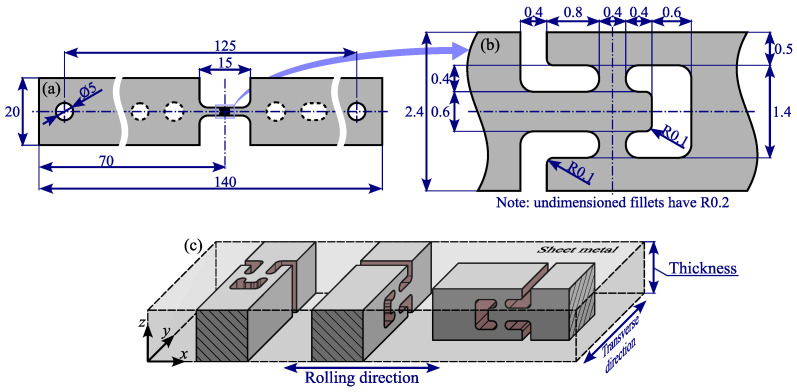
Schematic representation of the shear specimen geometry and orientation. (**a**) Overall dimensions of the shear specimen. (**b**) Detailed view of the shear bridge region with key dimensions. (**c**) A 3D illustration showing the positioning of the three shear details within the sheet metal, highlighting the orientation of shear detail to capture shear response in the x−y, y−z, and x−z material planes [37] (reproduced with permission from https://www.sciencedirect.com/journal/international-journal-of-solids-and-structures, accessed on 6 May 2025).

In Starman et al. [37], we developed a double-bridge out-of-plane shear test with pronounced sensitivity of the measured force response to variations in the Yld2004-18p out-of-plane shear parameters. The objective of this test is to conduct both in-plane and out-of-plane shear tests using the same shear detail geometry but oriented in different material planes. This approach enables a direct comparison of shear response differences across different material planes to unveil the differences in shear response in different planes. The test specimen design is presented in Figure 2.

In the study, we demonstrate that the proposed test exhibits improved resistance to shear zone rotation and ligament deformation, whereas an equivalent single-bridge test with the same effective shear bridge length exhibits significant shear zone rotation and ligament deformation.

The specimens were manufactured using Wire Electrical Discharge Machining (WEDM), resulting in a constrained design by WEDM manufacturing parameters (i.e., the cutting wire diameter) and the sheet thickness. While the wire diameter typically ranges from 0.05 to 0.3 mm, an additional clearance of 0.025 to 0.05 mm is automatically produced between the workpiece and the cutting wire. By analysing preliminary samples, we determined that the smallest reliably reproduced fillet was 0.2 mm, which led to a shear detail design incorporating 0.4 mm of the sheet’s thickness per shear bridge. Under this constraint, 1.62 mm of the thickness was left for ligament design. Our preliminary simulations confirmed that a ligament width of 0.5 mm is sufficient to prevent plastic deformation in the upper and lower ligaments in most cases. The remaining sheet width was utilised for the inner ligament width, as shown in Figure 2b.

**Figure 3:**
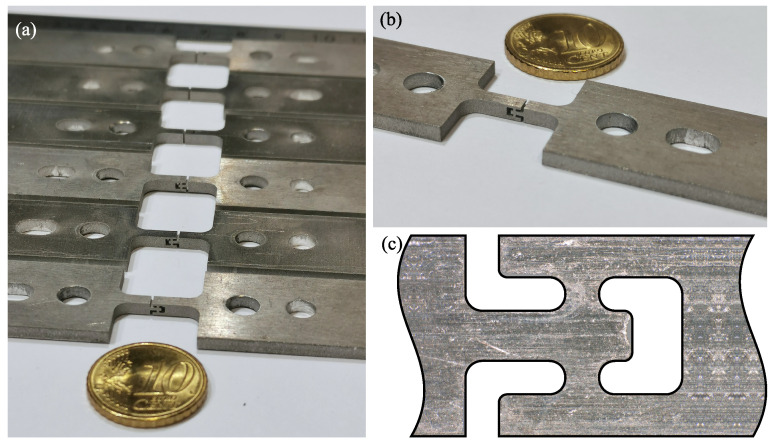
Images of the fabricated shear specimens. (**a**) A batch of shear specimens manufactured using WEDM. (**b**) Close-up view of a single shear specimen, highlighting the shear bridge details. (**c**) Magnified image of the shear bridge region [37] (reproduced with permission from https://www.sciencedirect.com/journal/international-journal-of-solids-and-structures, accessed on 6 May 2025).

When producing specimens of such small dimensions, it is essential to establish quality control and eliminate any samples where critical dimensions—such as shear bridge length, ligament width, and fillet radii—exceed the nominal values within the specified tolerance range. For this study, all produced samples were analysed using a Keyence VHX-6000 optical microscope (Osaka, Japan). To ensure consistent adherence to manufacturing tolerances, 10 samples per shear material plane were cut and examined. To minimise systematic errors, multiple measurements were taken from both sides of each sample. After statistical analysis, it was found that the mean shear bridge width was 0.388±0.010 mm, while the mean fillet produced was 0.206±0.005 mm. Finally, any samples exceeding these values were scrapped since they would potentially lead to biased measurements of shear response. The typically produced samples are presented in Figure 3.

### 2.4. Measurement of Shear Response

Before conducting tensile testing on the manufactured shear specimens, it is essential to analyse potential sources of systematic errors that may compromise the accuracy of DIC measurements. These errors may arise from the specimen manufacturing process, where deviations from the target dimensions occur; the specimen testing procedure, where asymmetric loading may cause bending; issues related to speckle pattern preparation and DIC settings, which can affect image processing and measurement accuracy; and variations in the local crystal structure, which may influence strain measurements.

When assessing such systematic errors, it is not only important to identify their origin but also to provide corrective measures for mitigation. In Starman et al. [37], we analysed the impact of manufacturing errors, such as asymmetric or oversized geometry (including non-symmetrical fillets, oversized shear bridges, and ligament height discrepancies), and found that these inconsistencies can be effectively eliminated by rejecting non-conforming specimens.

Furthermore, our study identified shear zone bending as the most dominant systematic error, occurring in two different planes. In-plane bending is caused by loading eccentricity, leading to asymmetric loading of the upper and lower bridge. This issue can be easily detected using 2D-DIC, as it results in different strain levels in the upper and lower ligament. If such asymmetry is observed during testing, it can be assessed by analysing strain differences between the upper and lower shear zones or ligaments. A more complex case arises when front–back bending occurs, where the front side of the specimen experiences greater stretching than the back side, distorting strain measurements on the front surface. Fortunately, this issue can be identified using 2D-DIC, as out-of-plane movement causes the specimen to go out of focus. Based on our recommendations, such defective measurements should be excluded from further analysis.

While our previous study [37] conducted a detailed sensitivity analysis of potential systematic error sources, DIC strain measurements were used primarily for sanity checks and to eliminate potentially corrupted data. Although in that study, the kinematics of the loaded specimens were monitored by measuring the effective extension of the shear detail using a virtual extensometer, the fundamental question regarding the accuracy and reliability of the strain field measurements remains unanswered.

#### 2.4.1. Test Setup and Loading Procedure

**Figure 4:**
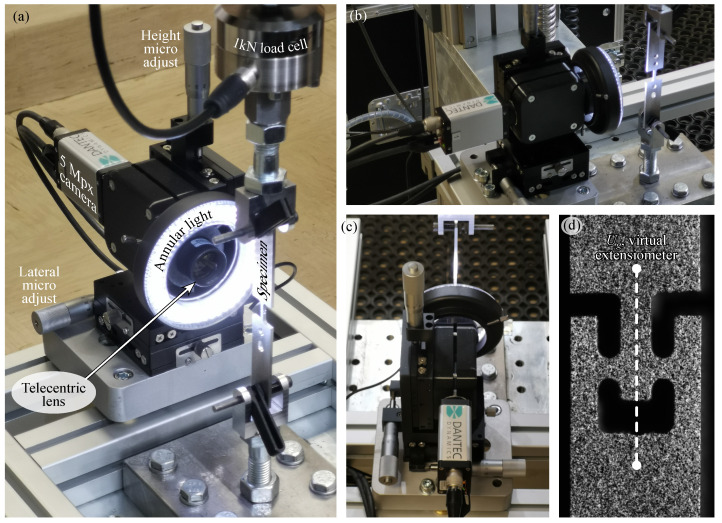
Experimental setup for shear testing with a DIC. (**a**) Close-up view of the optical measurement system, including a 5 Mpx camera, telecentric lens, and annular light source for high-resolution imaging. (**b**) Side view of the complete test setup, showing the specimen clamped in the loading fixture. (**c**) Top-down view of the optical system alignment with the specimen. (**d**) High-contrast speckle pattern applied to the shear zone for DIC measurements [37] (reproduced with permission from https://www.sciencedirect.com/journal/international-journal-of-solids-and-structures, accessed on 6 May 2025).

In this study, shear specimens were placed in a universal tensile tester equipped with custom-designed clevis grips. The specimens were secured by inserting pins through their outer holes, as illustrated in Figure 4a. Because the space between the clevis ligaments exceeded the sheet metal thickness, polyethylene spacers and springs were added to keep the specimen properly aligned in the pin direction, minimising bending caused by clamping. During setup, particular care was taken to assemble the clevises and mount them to the tester in a way that reduced loading eccentricity from external misalignments and avoided imposing any preload on the specimens. Testing proceeded under quasi-static conditions, with the cross-head moving at 0.1 mm/min. A 1 kN load cell (model µTC4, AEP transducers) recorded the force, and its output was synchronised with the speckle pattern image recording for DIC.

#### 2.4.2. Optical Setup and DIC System

A 2D-DIC system was employed to measure the displacement and strain fields in the shear region, as the region of interest demanded a relatively small field of view. The optical setup is depicted in Figure 4. A 5 Mpx digital camera (Manta G-201B, Allied Vision, Exton, PA, USA) recorded the speckled specimen under loading, and a telecentric lens (Coolens WWH15-63ATV3, Shenzhen Vico Technology Co., Shenzhen, China) provided a focused view of 5.6 mm× 4.7 mm around the shear zone (Figure 4d). Prior to testing, the system was calibrated using a 5 mm× 5 mm calibration target to correct for lens distortion. This camera-lens configuration offered high-resolution coverage of displacements and strains localised within a small area.

A key challenge involved maintaining a consistent focal setting. Minor misalignments during loading caused front-to-back bending of the specimen, and the telecentric lens’s small depth of field (approximately 0.3 mm) occasionally led to the camera losing focus. To mitigate this, the camera, lens, and lighting setup were secured to a linear translation stage with three micrometer screws, allowing fine adjustments in the xyz directions and ensuring proper focus throughout the experiment. Anyhow, when substantial front–back movement of the shear detail was noticed, this measurement was considered to be prone to systematic error.

The Istra4D software (ver 4.6.) provided continuous focus monitoring and interfaced with the DantecDynamic system for synchronised image capture and triggering. The entire DIC setup was rigidly attached to the universal testing machine, and an annular light source was employed for optimal illumination. It should be noted that numerical DIC parameters—such as the correlation algorithm, subset size, shape function, step size, interpolant, and strain window—significantly affect the accuracy of strain measurements and must be carefully chosen. Further details regarding these DIC parameters are listed in Table 1.

### 2.5. Direct-Levelling-Based Finite Element Model Updating

The classical FEMU is the most widely adopted full-field material identification strategy due to its intuitive foundation. In this approach, the mechanical test is numerically simulated using the finite element method (FEM), and the resulting kinematic field is compared with its counterpart measured by digital image correlation (DIC) [32]. The material model parameters in FEM can be refined by iteratively minimising the difference between DIC measurement and its counterpart in simulation. FEM and DIC fundamentally differ in how they compute the kinematic field, naturally leading to calibration error in FEMU due to the inconsistency of the strain field smoothness level. A levelling procedure is introduced to reduce inconsistencies between the FEM and DIC. While FEM analyses can be designed with sufficiently fine meshes to resolve strain gradients, DIC is limited by physical constraints on spatial resolution (e.g., camera detector size and lens optical resolution) at a desired strain uncertainty [38]. Furthermore, the DIC algorithm effectively acts as a low-pass filter, attenuating peak strain values. Consequently, “levelling” in this context refers to equalising some steps in FEM computations to account for the filtering effects of DIC. One way to accomplish this is by “pushing” FEM displacement data through the DIC processing chain: (i) synthetic deformed DIC images are generated by deforming the experimental reference speckle pattern images with the displacement fields simulated by FEM, (ii) the synthetic speckle pattern images and the experimental speckle pattern images are processed using DIC with the same parameter setting, and (iii) the resulting displacements are used to calculate strains via the so-called strain window method using the same virtual strain gauge (VSG). Known as DIC-levelling [42], this method effectively compensates for systematic errors such as filtering and image-induced artefacts. Assuming these artefacts are often negligible in the context of inverse calibration of a plasticity model, the procedure can be simplified by computing strains using the strain window method directly from FEM nodal displacements interpolated to the DIC data points. This streamlined method, referred to as direct-levelling, is computationally more efficient [38] and deemed sufficiently accurate for plasticity problems [43].

**Figure 5:**
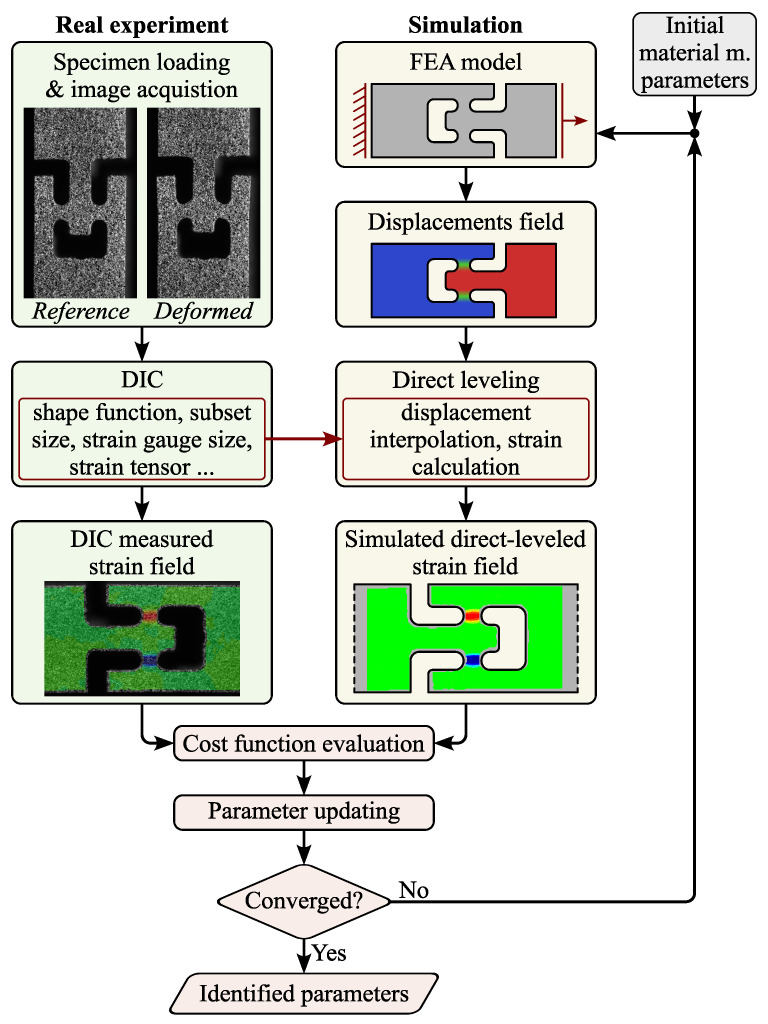
The FEMU procedure. The process integrates real experiments and numerical simulations to iteratively calibrate material parameters. On the experimental side, specimens are loaded, and deformation is captured using DIC, producing measured displacement and strain fields. In parallel, an FEM model is used to generate displacement fields and simulate strain fields through direct levelling. The difference between experimental and simulated strain fields is evaluated via a cost function, leading to updated parameters. This iterative process continues until convergence (evaluated by the cost function described in Equation (4)) is achieved, resulting in the identification of optimal material parameters.

For this particular shear test, the direct-levelling FEMU procedure is illustrated in Figure 5. The experiment follows the approach described in Section 2.4, where the DIC settings specified in Table 1 naturally introduce some filtering errors. To address these, the displacement fields obtained using the trial parameters $\alpha_{7},\alpha_{16}$ or $\alpha_{8},\alpha_{17}$ are converted to strains through the direct-levelling approach. Finally, the cost function is computed as described in the following subsection, and the parameters are updated using the *Levenberg–Marquardt* algorithm. Naturally, the FEMU procedure is then repeated independently for both the x−z and y−z material planes.

#### Cost Function and Parameter Updating

The basic idea behind the FEMU procedure is to minimise the difference between the strain fields measured experimentally (via DIC) and those obtained numerically. Here, the only dominant strain component—which also directly correlates with the sought parameters—is $\varepsilon_{xz}$ or $\varepsilon_{yz}$. Therefore, the FEMU optimisation is performed by solving

(4) $$\min_{p_{q}}C\left(p_{q}\right)=\frac{1}{2}\sum_{i=1}^{n}{\left(\frac{\varepsilon_{q,i}^{\mathrm{num}}\left(p_{q}\right)-\varepsilon_{q,i}^{\exp}}{\varepsilon_{q}^{\mathrm{RMS}}}\right)}^{2},$$

where q∈{xz,yz} denotes either the x−z or y−z material plane, respectively. The parameters $p_{xz}={\left\{\alpha_{7},\alpha_{16}\right\}}^{T}$ and $p_{yz}={\left\{\alpha_{8},\alpha_{17}\right\}}^{T}$ are identified through two separate optimisation processes. The cost function $C(p_{q})$ focuses on minimising the difference between the experimentally measured $\varepsilon_{q}^{\exp}$ and the numerically simulated $\varepsilon_{q}^{\mathrm{num}}$ logarithmic shear strain at each ith point, out of *n* data points in the region of interest (ROI) at maximum load. Lastly, the cost function is normalised by the root mean square (RMS) of the experimental data, calculated as $\varepsilon_{q}^{\mathrm{RSM}}=\sqrt{\frac{1}{n}\sum_{i=1}^{n}{\left(\varepsilon_{q,i}^{\exp}\right)}^{2}}$.

To solve the least-squares problem introduced by Equation (4), the *Levenberg–Marquardt* method was applied, where the parameters $p_{q}$ are updated in each kth iteration according to

(5) $$p_{q}^{\left(k+1\right)}=p_{q}^{\left(k\right)}+{\left(S^{T}S+\lambda I\right)}^{-1}S^{T}\left(\varepsilon_{q}^{\mathrm{num}}\left(p_{q}^{\left(k\right)}\right)-\varepsilon_{q}^{\exp}\right).$$

Here, $\varepsilon_{q}^{\mathrm{num}}\left(p_{q}^{\left(k\right)}\right)$ and $\varepsilon_{q}^{\exp}$ represent the normalised global strain vectors, assembled from the shear strain component at all measurement points. The matrix S is the (n×2)-dimensional strain sensitivity matrix, defined as $S={\left.\partial \varepsilon_{q}^{\mathrm{num}}\left(p_{q}\right)/\partial p_{q}\right|}_{\left(k\right)}$, which is computed numerically using the forward difference scheme. The symbol λ is known as the damping factor, and I is a (2×2)-dimensional identity matrix. As indicated, the numerical values $\varepsilon_{q}^{\mathrm{num}}$ are calculated using the current parameter estimates $p_{q}^{\left(k\right)}$.

We used our in-house FEMU software platform FEMid (ver 1.2) [44] to process the simulated data from ABAQUS (ver 2024) and the measured data from MatchID-2D (ver 2024.2.3). In the software, the FEM model provides the nodal displacement field on the specimen surface, while the DIC code supplies the displacement field following subset-based correlation. The strain window method is then applied to both displacement fields to obtain the strains at each DIC data point. Finally, the optimisation is deemed convergent when the relative change in the cost function falls below $10^{-4}$.

## 3. Results

The identification of out-of-plane shear parameters for the Yld2004-18p model generally requires an experimental response, against which the model response is compared and iteratively updated to minimise discrepancies. For this procedure to be meaningful, certain model parameters—such as those governing hardening behaviour and the reference direction for that hardening—must be predefined.

Conventionally, the specified hardening curve is based on uniaxial tensile test data in the rolling direction, which implies that the in-plane anisotropy parameters must also be determined from uniaxial and equi-biaxial test data. Since this procedure is well-documented in the literature (e.g., [10]) and the in-plane parameters for the particular material model and sheet metal have already been established, the detailed description of their derivation is omitted here for brevity. The complete procedure for determining the in-plane parameters from standard tests on this specific sheet metal is provided in Starman et al. [37]. The results of standard testing and the corresponding in-plane parameters $\alpha_{1},\alpha_{2},\ldots ,\alpha_{6}$, $\alpha_{9},\ldots ,\alpha_{15}$, and $\alpha_{18}$ are summarised in Table 2. The out-of-plane parameters $\alpha_{7},\alpha_{8},\alpha_{16}$, and $\alpha_{17}$ are considered unknown and are set to one as an initial trial.

### 3.1. Virtual Experimentation

**Figure 6:**
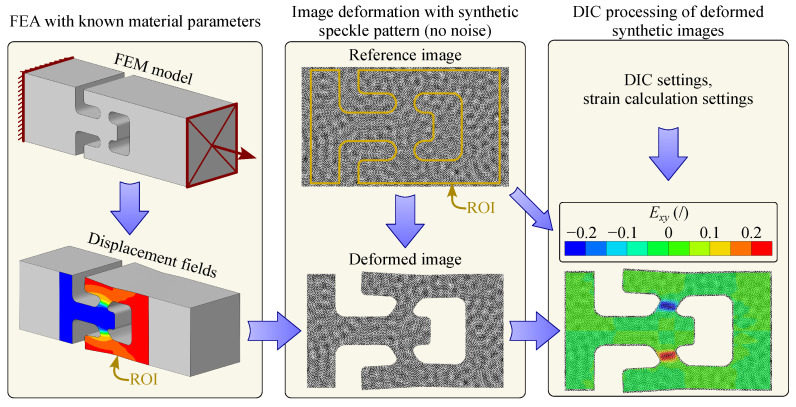
The concept of virtual experimentation. The process begins with an FEM model using known material parameters to generate displacement fields. These displacement fields are then applied to a synthetic speckle pattern to create a reference and a deformed image, simulating real experimental data. The deformed synthetic images are subsequently processed using a DIC algorithm, where strain fields are computed based on DIC and strain calculation settings. This virtual approach enables a validation of the FEMU optimisation procedure before conducting an identification on real measured data.

Before conducting an inverse identification, it is crucial to examine the influence of DIC settings on the strain field reconstruction, as well as to verify the implemented direct-levelling-based FEMU identification chain. To demonstrate the validity of this procedure, a virtual experiment is performed. Specifically, a numerical simulation of the model response is carried out using known material parameters. The resulting displacement fields are then used to synthetically deform an artificially generated or real, undeformed speckle pattern via an image deformation process, producing numerically generated deformed images that ideally mirror the simulated state. In the subsequent step, these deformed images—treated as if they were actual camera acquisitions—are fed into the DIC code to reconstruct the displacement fields and the strain field using a virtual strain gauge approach. While this procedure is equivalent to the DIC-levelling method, it yields fields using the same procedure as the DIC engine. The concept of virtual experimentation is illustrated in Figure 6.

To verify the implemented direct-levelling-based FEMU procedure, the previously processed fields replace the measured data from a real experiment during the inverse identification scheme in Figure 5. Finally, the case showed that the procedure was able to reconstruct the target parameters with good accuracy.

### 3.2. The Measured Shear Response

Eight specimens were tested for each shear plane using the experimental setup described in Section 2.4. It should be emphasised that only consistently manufactured test specimens were selected for testing.

After discarding measurements potentially affected by systematic errors following the methods described in Section 2.4, approximately half of the initially prepared samples were retained for further analysis. In addition, to demonstrate differences in the measured characteristic responses in different shear planes, a virtual extensometer was placed at the centre of the specimen. Because the bulkier sections of the specimen primarily undergo rigid body motion, the measured extension mainly reflects the extension of the grips.

**Figure 7:**
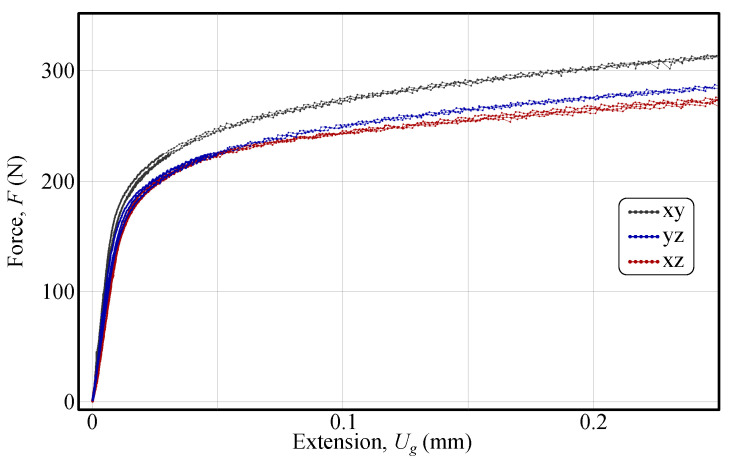
Force–extension curves for different shear detail orientations. The plot presents the force *F* (N) as a function of extension $U_{g}$ (mm) for three shear detail orientations: x−y (black), y−z (blue), and x−z (red). Differences in the force response among the orientations highlight the anisotropic shear behaviour of the material [37] (reproduced with permission from https://www.sciencedirect.com/journal/international-journal-of-solids-and-structures, accessed on 6 May 2025).

The force–extension curves for all three tested shear planes are shown in Figure 7. A key observation is that the initial linear elastic response fairly aligns across all curves, indicating that the samples have similar stiffness, thereby confirming the consistency of the manufacturing process. This finding also validates the procedure used to exclude specimens prone to loading eccentricity due to misalignment. Furthermore, as presented, the highest force response occurs in the x−y material plane, whereas the y−z and x−z planes exhibit responses that are approximately 8% and 12% lower, respectively.

### 3.3. Calibration of Out-of-Plane Anisotropy

The calibration of the out-of-plane shear parameters $\left\{\alpha_{7},\alpha_{16}\right\}$ and $\left\{\alpha_{8},\alpha_{17}\right\}$ was carried out in two separate identification processes. For each identification, the experimental result that closely matched the mean response in Figure 7 was selected and post-processed to obtain the experimental displacement field. Next, the measured geometry of both samples was modelled and simulated in ABAQUS to capture the impact of manufacturing, and the displacement field was imported into FEMid for post-processing in each iteration. It should be emphasised that data points in the ROI with an equivalent von Mises strain of less than 0.02 were excluded from the cost function. A NICE scheme was employed to integrate the Yld2004-18p material model [45,46]. It should be emphasised that force-driven loading conditions were used in the simulations to ensure consistency with the experiment and to guarantee that the simulated and measured force responses coincided. When post-processing the simulated and measured displacement fields, the same DIC settings specified in Table 1 were used. As in the virtual experiment, the initial values of the sought parameters were set to one. The results of the identified parameters with their corresponding evolution are presented in Table 3.

**Figure 8:**
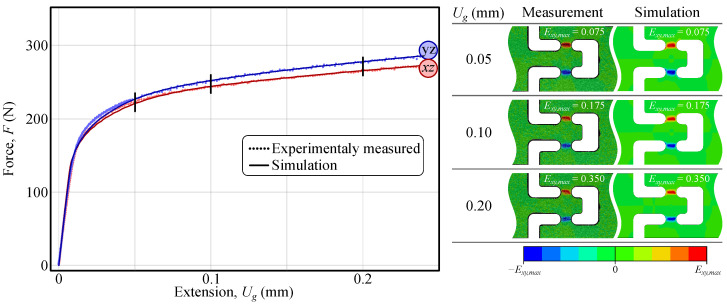
Comparison of the force–extension and full-field strain response between experimental measurements and numerical simulations using the identified material parameters (Table 3). The left plot shows force *F* versus extension $U_{g}$, with experimentally measured data (dotted lines) compared against simulation results (solid lines) for two shear orientations (y−z and x−z). The right section presents strain field comparisons at different extension levels ($U_{g}$ = 0.05, 0.10, and 0.20 mm), illustrating good agreement between the DIC measurements and the FEM predictions [37] (reproduced with permission from https://www.sciencedirect.com/journal/international-journal-of-solids-and-structures, accessed on 6 May 2025).

The validity of the identified material parameters was confirmed by the close agreement between the simulated and experimentally measured strain fields (Figure 8, right). It is important to note that in the actual experiment, the samples were subjected to displacement-driven rather than force-driven loading conditions. Consequently, both calibration approaches are expected to yield equivalent displacement and strain fields, as the input forces used in the simulations correspond to the imposed displacements. For completeness, the force–extension response was also simulated for the y−z and x−z material planes and compared with the experimental measurements. The results are shown in Figure 8.

**Figure 9:**
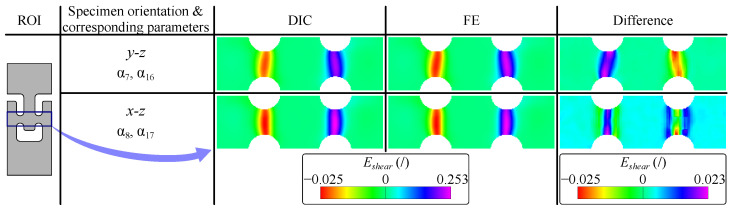
Comparison of measured and identified shear strain fields with corresponding difference.

As shown in Figure 9, the measured and simulated shear strain fields are closely aligned, with discrepancies limited to approximately 2%. Furthermore, comparison of the values of the identified parameters with those obtained using a conventional force–elongation curve-based approach [37] (Table 4) reveals a difference in parameters of less than 4%. This indicates that the out-of-plane shear behaviour can be accurately characterised using the measured strain fields, which reflect the overall specimen response rather than being dominated by local grain-level effects.

## 4. Discussion

This paper presents a full-field, macro-mechanical methodology for characterising the out-of-plane shear anisotropy of medium-thick sheet metals using a double-bridge shear specimen. The key idea of the shear test is to employ a double-bridge specimen design with identical shear detail dimensions in all three material planes. In this way, the shear responses in the x−y, x−z, and y−z planes can be measured and directly compared. Additionally, the test design combined with DIC allows direct detection of undesired specimen bending, in which the upper and lower shear bridges deform differently. Such measurements, together with the specimens of insufficient manufacturing quality, were excluded from further analyses.

Displacements and strains in the shear regions were measured using 2D-DIC. Beyond the possible technical challenge of losing focus, a significant limitation in evaluating micro-shear strains is the local structure of the material, which governs the strain state at the specimen’s surface and may differ from the representative value through the thickness of the shear detail. Secondly, as the strains are largely localised within a confined region of the shear bridges, the measured shear strain is, to some extent, averaged by DIC filtering, meaning the actual peak strains in these zones are higher. This was confirmed by a virtual experiment, in which the simulations generally yielded higher strains than DIC measurements, depending on the strain window size.

**Figure 10:**
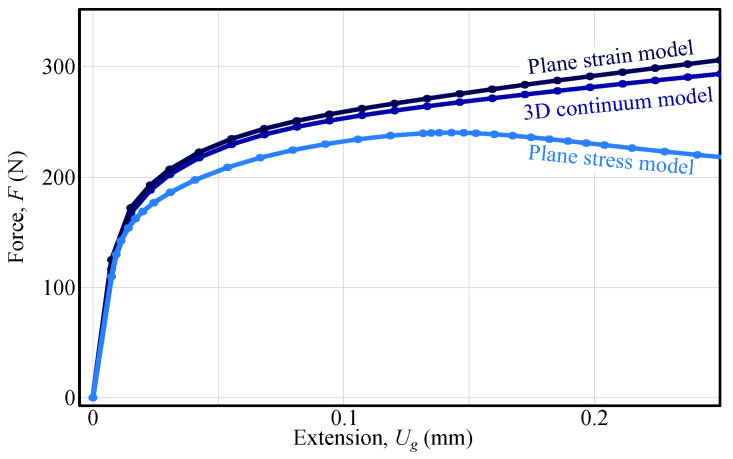
Comparison of force-extension curves for different numerical modelling approaches. The plot presents force *F* versus shear detail elongation $U_{g}$ for three modelling assumptions: plane strain model (dark blue), 3D continuum model (blue), and plane stress model (light blue). The results highlight the influence of stress state assumptions on the predicted mechanical response, with the plane strain model exhibiting the highest force response and the plane stress model the lowest.

Nevertheless, a simple comparison of different modelling assumptions can be used to analyse whether the measured surface strains are constant through the thickness of the specimen. While a 3D continuum model of the shear specimen represents the most realistic configuration, it is worth asking how accurately plane–strain or plane–stress assumptions capture the specimen’s behaviour. To investigate this, both models were built and analysed in ABAQUS, and the resulting force–extension curves are shown in Figure 10. As illustrated, the plane–strain model response closely follows that of the 3D continuum model, whereas the plane–stress model undergoes strain localisation, resulting in a drop in force.

From the results, it can be concluded that under the plane–strain assumption, the measured surface strains are roughly representative of a complete continuum, provided there is no front–back bending of the specimen. Furthermore, when a 3D continuum model is used in FEMU, the spatial variation of strains is inherently captured during identification.

Finally, it should be noted that the differences in out-of-plane shear anisotropy are relatively small (8% and 12% lower response in the y−z and x−z planes compared to the x−y plane), making the applicability of a plane–strain model questionable. In particular, for inverse identification strategies such as the conventional non-linear Virtual Fields Method (VFM), the specimen would need to be thinner or wider to satisfy plane–stress or plane–strain conditions. However, even if this can be experimentally achieved, the issue of localised strain gradients persists. While FEMU pragmatically handles this through levelling approaches, such an option is not currently available in VFM, meaning that the presented shear test design is not compatible with VFM. Finally, it can be concluded that the developed FEMU provides a robust framework for identifying material behaviour from the double-bridge shear experiment.

## 5. Conclusions

This paper presents a novel FEMU-based approach for the characterisation of through-thickness plastic shear anisotropy of sheet metal. The key findings and contributions of this study are summarised as follows:

- Compared to existing specimen designs, the proposed shape leverages the advantages of double-bridge shear testing, increasing the sensitivity of the measured response to perturbations in out-of-plane shear parameters.
- The consistent geometry of the shear detail across all sheet orientations allows for direct comparison of shear responses in different material planes.
- The influence of specimen manufacturing precision on measurement accuracy was thoroughly investigated. Wire electrical discharge machined specimens were analysed for key dimensional features, and the actual geometry was incorporated into the identification process. Among the evaluated error sources, eccentric loading—inducing front-to-back bending—was found to have the most significant impact, leading to distorted surface strain measurements.
- The testing procedure employs a high-resolution 2D-DIC setup, incorporating a 5 MPx camera with a telecentric lens and annular light source. The strain field is measured and subsequently processed using subset-based DIC.
- The FEMU inverse identification scheme integrates full-field strain data, enabling independent parameter identification across different material planes.
  – A key feature of the identification scheme is the intrinsic consideration of local strain gradients from the FEM analysis, which are directly accounted and levelled using the strain window method. This ensures that the FEM-computed and DIC-processed strains undergo the same DIC post-processing procedure, effectively eliminating systematic error. In contrast, other methods, such as VFM, require spatially converged strain fields.
  – The FEM model inherently captures critical multiaxial stress–strain behaviour, which is essential for accurate inverse identification. This confirms that the mechanical response cannot be adequately described by either plane stress or plane strain assumptions.
  – Incorporating measured strain fields into the FEMU identification framework disproves the hypothesis that the surface strain responses are not representative of the specimen’s global behaviour.
  – Parameter values identified using the proposed method are in close agreement with those obtained from traditional force–extension curve-based identification, as demonstrated in Starman et al. [37].
- For a 2.42 mm thick cold-rolled AW5754-H22 aluminium alloy sheet, force–extension responses measured in the y−z and x−z planes were found to be 8% and 12% lower, respectively, compared to those in the x−y plane, indicating the presence of through-thickness shear anisotropy.

## Figures and Tables

**Table 1:** Equipment and DIC settings used in the experiment.

Equipment, Settings	Specifications
Camera	Manta G-507B, Allied Vision, (Exton, PA, USA)
Telecentric lens	Coolens WWH15-63ATV3
Image resolution	2464 × 2056 px
Telecentric lens working distance	63 mm
Telecentric lens depth of field	0.3 mm
DIC technique	2D DIC
DIC software	MatchID 2024.2.3, DantecDynamics Istra 4D (ver 4.6.)
Illumination colour temp.	5600 K
Patterning technique	white surface with black speckles (airbrush)
Region of Interest (ROI)	2.5 × 5 mm
Pixel to mm conversion	1 px = 2.3 µm
Average speckle size	12.8 px
Image filtering	gaussian, 5 × 5 px
Subset size	21 × 21 px
Step size	5 px
Subset weight	uniform
Subset shape function	affine
Matching criterion	approximated sum of squared differences
Interpolant	local bicubic spline interpolation
Reference image	fixed
Data points	11,700
Deformation gradient	strain window size: 7 × 7 data points interpolation: Bilinear Quadrilateral (Q4)
Temporal smoothing	none
Acquisition frequency	10 Hz
Cross-head speed	0.1 mm/min
Virtual strain gauge size	0.232 × 0.232 mm

**Table 2:** Measured normalised yield stresses and Lankford coefficients of the AW5754-H22 aluminium alloy. The identified in-plane $\alpha_{1},\alpha_{2},\ldots ,\alpha_{6}$, $\alpha_{9},\ldots ,\alpha_{15}$, and $\alpha_{18}$ parameters of the Yld2004-18p model are given in the right-hand side of the table.

Parameter	Value [-]	Parameter	Value [-]	Parameter	Value [-]	Parameter	Value [-]
$Y_{0{}^{\circ}}$	1	$r_{0{}^{\circ}}$	0.47	$\alpha_{1}$	1	$\alpha_{10}$	1.166
$Y_{11.25{}^{\circ}}$	0.990	$r_{11.25{}^{\circ}}$	0.46	$\alpha_{2}$	1	$\alpha_{11}$	1.117
$Y_{22.5{}^{\circ}}$	0.980	$r_{22.5{}^{\circ}}$	0.49	$\alpha_{3}$	0.090	$\alpha_{12}$	0.881
$Y_{33.75{}^{\circ}}$	0.952	$r_{33.75{}^{\circ}}$	0.64	$\alpha_{4}$	0.507	$\alpha_{13}$	1.024
$Y_{45{}^{\circ}}$	0.932	$r_{45{}^{\circ}}$	0.75	$\alpha_{5}$	1.069	$\alpha_{14}$	−0.491
$Y_{56.25{}^{\circ}}$	0.951	$r_{56.25{}^{\circ}}$	0.80	$\alpha_{6}$	1.127	$\alpha_{15}$	1.144
$Y_{67.5{}^{\circ}}$	0.964	$r_{67.5{}^{\circ}}$	0.72	$\alpha_{9}$	−0.779	$\alpha_{18}$	1.295
$Y_{78.75{}^{\circ}}$	0.977	$r_{78.75{}^{\circ}}$	0.75				
$Y_{90{}^{\circ}}$	0.984	$r_{90{}^{\circ}}$	0.77	*E* [GPa]	70.3	ν [–]	0.33

**Table 3:** Evolution of optimised Yld2004-18p out-of-plane shear parameters ($\alpha_{7}$, $\alpha_{8}$, $\alpha_{16}$, $\alpha_{17}$) and corresponding normalised objective function values ($\bar{C}\left(p_{yz}\right)$, $\bar{C}\left(p_{xz}\right)$) throughout the identification procedure. The final, optimised parameter values are highlighted in bold.

$\alpha_{8}$ [-]	$\alpha_{17}$ [-]	$\bar{C}\left(p_{\mathrm{yz}}\right)$ [-]	$\alpha_{7}$ [-]	$\alpha_{16}$ [-]	$\bar{C}\left(p_{\mathrm{xz}}\right)$
1	1	1	1	1	1
0.7827	1.4913	0.8836	0.9442	1.3983	0.8271
0.4484	1.7208	0.3817	1.0357	1.2678	0.5259
0.4626	1.6743	0.2880	1.1932	1.0801	0.3396
0.4651	1.6736	0.2878	1.0411	1.2223	0.2442
0.4678	1.6724	0.2872	1.0779	1.1841	0.2377
0.4705	1.6712	0.2868			
0.4748	1.6635	0.2711			
**0.4748**	**1.6635**	**0.2711**	**1.0779**	**1.1841**	**0.2377**

**Table 4:** Comparison of identified out-of-plane shear parameters using FEMU and classical force–extension-based identification, including their relative differences.

	$\alpha_{8}$ [-]	$\alpha_{17}$ [-]	$\alpha_{7}$ [-]	$\alpha_{16}$ [-]
FEMU identification	0.475	1.664	1.078	1.184
Force–extension-based [37]	0.494	1.661	1.060	1.168
Relative difference [%]	3.89	0.15	1.69	1.38

## Data Availability

The original contributions presented in this study are included in the article. Further inquiries can be directed to the corresponding authors.
